# Cross‐Culturally Translating and Validating a Dementia Attitudes Scale in Arabic, Vietnamese, Cantonese, Mandarin, and Greek

**DOI:** 10.1002/alz.091704

**Published:** 2025-01-09

**Authors:** Diana Karamacoska, Nibras Jasim, Mai Tran, Gabriela E Caballero, Emy Tsouros, Sonia Chan, Eman Shatnawi, Genevieve Z Steiner‐Lim, Joyce Siette, Eliza Chan, Kim‐Huong Nguyen, Hanh Dao Tran

**Affiliations:** ^1^ NICM Health Research Institute, Western Sydney University, Sydney, NSW Australia; ^2^ Translational Health Research Institute, Western Sydney University, Penrith, NSW Australia; ^3^ Western Sydney University, Sydney, NSW Australia; ^4^ MARCS Institute for Brain, Behaviour and Development, Western Sydney University, Penrith, NSW Australia; ^5^ Australian Nursing Home Foundation, Sydney, NSW Australia; ^6^ University of Queensland, Brisbane, QLD Australia

## Abstract

**Background:**

Dementia is highly stigmatised, misperceived as a mental illness, and considered a normal part of ageing by people from culturally and linguistically diverse backgrounds in Australia. There is a lack of valid and reliable scale to measure their dementia attitudes. This study aimed to cross‐culturally translate and validate a dementia attitudes scale in Arabic, Vietnamese, Chinese, and Greek communities as they represent the main languages spoken throughout Western Sydney, Australia.

**Method:**

A 12‐item Australian dementia attitudes scale, initially adapted from the PRISM‐PC–Dementia Screening Subscale, underwent forward and backward translation in the Arabic, Vietnamese, Cantonese, Mandarin, and Greek languages with certified translators and bilingual project team members. The translated surveys were also piloted with native speaking older community members to resolve comprehension issues. The final version of each scale was distributed online and at community events. Scale data (Arabic *N* = 197; Vietnamese *N* = 121; Cantonese *N* = 107; Mandarin *N* = 219; Greek *N* = 1,050) underwent reliability testing (Cronbach’s α) and exploratory factor analysis (EFA) with principal axis factoring and Promax rotation.

**Result:**

The translated scales demonstrated high reliability across all five language groups (Cronbach α ≥ .77). The EFAs revealed distinct factor solutions for the Arabic, Vietnamese, Cantonese, and Mandarin scales, with ‘fear of labelling’ emerging as a common stigma‐related factor. Ten of the twelve items in the Greek scale did not meet retention criteria, limiting the scale’s validity.

**Conclusion:**

The cross‐cultural translation procedures yielded reliable scale outcomes across the five languages. The distinct factor solutions for each scale highlight the impact of culture on the stigma toward dementia. These scales can help researchers and community organisations efficiently explore dementia attitudes in under‐served populations and tailor education programs to combat misperceptions. However, a more robust scale for the Greek speaking community needs to be developed.

